# Correction: Huang et al. Systemic Anticoagulation and Inpatient Outcomes of Pancreatic Cancer: Real-World Evidence from U.S. Nationwide Inpatient Sample. *Cancers* 2023, *15*, 1985

**DOI:** 10.3390/cancers16061181

**Published:** 2024-03-18

**Authors:** Yen-Min Huang, Hsuan-Jen Shih, Yi-Chan Chen, Tsan-Yu Hsieh, Che-Wei Ou, Po-Hsu Su, Shih-Ming Chen, Yun-Cong Zheng, Li-Sung Hsu

**Affiliations:** 1 Hemophilia and Thrombosis Treatment Center, Division of Hematology and Oncology, Department of Internal Medicine, Keelung Chang Gung Memorial Hospital, Keelung 204, Taiwan; 8902004@cgmh.org.tw (Y.-M.H.); b9505015@cgmh.org.tw (P.-H.S.); 2 Division of Hematology and Oncology, Department of Internal Medicine, Lotung Poh-Ai Hospital, Yilan 265, Taiwan; 3 Institute of Medicine, Chung Shan Medical University, Taichung 402, Taiwan; 4 Division of Hematology and Oncology, Department of Internal Medicine, Linkou Chang Gung Memorial Hospital, Taoyuan 333, Taiwan; samuel.shih@outlook.com (H.-J.S.); cheweiou@gmail.com (C.-W.O.); 5 Department of General Surgery, Keelung Chang Gung Memorial Hospital, Keelung 204, Taiwan; a017749@gmail.com; 6 Department of Pathology, Keelung Chang Gung Memorial Hospital, Keelung 204, Taiwan; 8902059@cgmh.org.tw; 7 Bachelor Program in Health Care and Social Work for Indigenous Students, Providence University, Taichung 433, Taiwan; big32762992@pu.edu.tw; 8 Departments of Neurosurgery, Keelung Chang Gung Memorial Hospital, Keelung 204, Taiwan; 9 College of Medicine, Chang Gung University, Taoyuan 333, Taiwan; 10 Department of Biomedical Engineering, National Taiwan University, Taipei 110, Taiwan; 11 Department of Medical Research, Chung Shan Medical University Hospital, Taichung 402, Taiwan

## 1. Additional Affiliation(s)

In the original publication [1], there was an error regarding the affiliation**(s)** for “**Li-Sung Hsu**”. In addition to affiliation(s) “**3**”, the updated affiliation(s) should include: “**Department of Medical Research, Chung Shan Medical University Hospital, Taichung 402, Taiwan**” as affiliation “**11**”. The authors state that the scientific conclusions are unaffected. This correction was approved by the Academic Editor. The original publication has also been updated.
